# Actual Results of the Test

**Published:** 1914-05-02

**Authors:** 


### Actual Results of the Test.

As far as the preliminary report of this research goes, the number of cases experimented on is much too small for hard-and-fast conclusions to be laid down concerning the accuracy of this reaction. Clearly enough, even an inaccuracy of 1 or 2 per cent. (such as exists in Widal's test for typhoid fever) would impair this reaction to such an extent as to make it useless. The authors' cases were as follows: A positive reaction in three pregnant married women, when injected with blood corpuscles from their respective husbands. One of these women was also injected with corpuscles from a stranger, with negative results. Thirteen pregnant women were then injected with corpuscles from men who were strangers to them: twelve results were negative and one was doubtful. Corpuscles from two husbands gave positive reactions when injected into their respective pregnant wives, but no reactions with three other pregnant women whom they had never known. In five non-pregnant women, who had previously borne their husbands children, there were negative results in four and a doubtful reaction in one. This reaction, so interesting in the possibilities it opens up of the future of hæmopathology, will be recognised as merely an extension of the principle of Elsberg's cancer reaction. It is some months now since the original paper appeared; and this interval may possibly mean that the test is less exact than was at first thought.

